# Screening of cold hardiness-related indexes and establishment of a comprehensive evaluation method for grapevines (*V. vinifera*)

**DOI:** 10.3389/fpls.2022.1014330

**Published:** 2022-11-24

**Authors:** Zhi-Lei Wang, Dong Wu, Miao Hui, Ying Wang, Xing Han, Fei Yao, Xiao Cao, Yi-Han Li, Hua Li, Hua Wang

**Affiliations:** ^1^ College of Enology, Northwest A&F University, Yangling, Shaanxi, China; ^2^ College of Life Science, Langfang Normal University, Langfang, Hebei, China; ^3^ Shaanxi Engineering Research Center for Viti-Viniculture, Yangling, Shaanxi, China; ^4^ China Wine Industry Technology Institute, Yinchuan, Ningxia, China; ^5^ Engineering Research Center for Viti-Viniculture, National Forestry and Grassland Administration, Yangling, Shaanxi, China

**Keywords:** grapevine, cold hardiness, physiological and biochemical indexes, comprehensive evaluation, *V. vinifera* germplasm resources

## Abstract

The goals of this work were to screen physiological and biochemical indexes to assess a set of *V. vinifera* germplasm resources, to compare evaluation methods for cold hardiness, and to establish a comprehensive method that can be used for more accurate screening for cold hardiness in *V. vinifera.* Four single methods were used to evaluate the cold hardiness of 20 germplasms resources and 18 physiological and biochemical indexes related to cold hardiness were determined. The LT_50_ values determined by electrical conductivity (EL), 2,3,5-triphenyltetrazolium chloride staining (TTC), differential thermal analysis (DTA), and recovery growth (RG) methods showed extremely significant positive correlation. Bound water content (BW), proline content (Pro), total soluble sugar content (TSS), malondialdehyde content (MDA), catalase content (CAT), and ascorbic acid content (ASA) exhibited significant correlation with LT_50_ values measured by different evaluation methods. The comprehensive cold hardiness index calculated by principal component analysis (PCA) combined with subordinate function (SF) was negatively correlated with LT_50_ values measured by different evaluation methods. Meili and Ecolly exhibited the highest cold hardiness, indicating their potential for use as parents for cold hardiness breeding. EL, DTA, TTC, and RG methods successfully distinguished cold hardiness among different *V. vinifera* germplasm lines. Measurements of BW, Pro, TSS, MDA, CAT, and ASA in dormant shoots also can be used as main physiological and biochemical indexes related to cold hardiness of *V. vinifera*. Comprehensive evaluation by PCA combined with SF can accurately screen cold hardiness in *V. vinifera*. This study provides a reference and accurate identification method for the selection of cold hardiness parents and the evaluation of cold hardiness of germplasm of *V. vinifera*.

## 1 Introduction

As a main cultivar, *V. vinifera* grapes have extremely high economic value and are used to produce grapes consumed fresh or dried or used to make wine or juice. *V. vinifera* varieties are often used for as starting material to generate improved varieties and as parent materials for high-quality hardiness breeding (Wang et al., 2021b). However, the mature shoots of *V. vinifera* can only tolerate a low temperature of about -15°C during winter dormancy, so in some cold regions, such as northern China and the Russian far East, soil-burial over-wintering of vines has become the main cultivation mode of *V. vinifera* (Wang et al., 2021b). Although effective, this practice is labor intensive, thus restricts the sustainable development of viticulture (Han et al., 2021). Therefore, it is important to breed cold-resistant new varieties that can be cultivated in these regions without the requirement for vine burial during winter. To successfully breed cold resistant varieties, effective methods are required to evaluate cold hardiness.

There have been many studies of the physiological and biochemical aspects of grapevine cold hardiness and different methods have been proposed to evaluate cold hardiness (Avanci et al., 2010; Jeon et al., 2010; Jin et al., 2012; Zhang F. et al., 2012; Hu et al., 2013). The classical evaluation method of grapevine cold hardiness uses electrical conductivity (EL) to measure the electrolyte leakage of shoot tissues at low temperatures (Sutinen et al., 1992; Elisa et al., 2009; Wang et al., 2021b). The semi-lethal temperature (LT_50_) can then be calculated from these measurements using the logistic equation (Elisa et al., 2009). Another technique, differential thermal analysis (DTA), detects and records the exothermic process at low temperature, and then analyzes and evaluates the cold hardiness of plant tissues (Chai et al., 2015; Kaya and Köse, 2017; Londo and Kovaleski, 2017; Muhammed and Cafer, 2021). The DTA method has been applied to the study of cold hardiness of grape shoots, roots, and buds (Chen et al., 2020a). The tissue browning method is another objective and reliable method used to study cold hardiness of grapevine (Niu and He, 1993). In this method, the browning of shoot slices is observed under a microscope after freezing and classifying freezing damage based on the browning area of secondary xylem (Niu and He, 1993). Another method uses 2,3,5-Triphenyltetrazolium chloride (TTC) staining to measure tissue viability and cold hardiness of plants (Thomas and Ahlers, 2010). Zhao et al. successfully identified the cold hardiness of *V. amurensis* using TTC staining index and logistic equation (Zhao et al., 2018). Restoration growth (RG) may be the most intuitive and reliable method of cold hardiness measurement of grapevine as it directly observes the germination, rooting, and callus production rates after low temperature treatment (Fennell, 2004; Chen et al., 2014; Liu Z. et al., 2021).

There are many *V. vinifera* cultivars and the cold hardiness of different varieties varies, with few *V. vinifera* varieties are suitable for cultivation in any specific country or region (Zhan and Li, 2010; Wang et al., 2021b). Most research on *V. vinifera* has focused on cultivation management (Ju et al., 2019; Yue et al., 2020; Wang et al., 2021a; Yue et al., 2021), fruit quality regulation (Bohan et al., 2020; Li et al., 2021; Yang N. et al., 2021), wine-making characteristics (Sun et al., 2021; Wei et al., 2022), grape and wine nutrition (Cheng et al., 2021; Xu et al., 2021; Yang C. et al., 2021), and hardiness improvement (Liu R. et al., 2021; Wan et al., 2021; Wang X. et al., 2021; Wang et al., 2021c; Wang et al., 2022), but there has been little comprehensive work to determine the most appropriate methods for the evaluation of cold hardiness in *V. vinifera*. Most methods used thus far to investigate cold hardiness are relatively simple methods that are not targeted, so the results obtained using different cold hardiness evaluation methods will be different (He, 2015). This lack of comprehensive and effective evaluation limits the full utilization of *V. vinifera* germplasm resources.

The goal of this study was to test the effectiveness of four cold hardiness evaluation methods to screen the cold hardiness of 20 V*. vinifera* lines. The correlations between various related physiological and biochemical indexes and cold hardiness were analyzed and indexes of cold hardiness were screened, allowing the establishment of a comprehensive evaluation method for cold hardiness in *V. vinifera.* The results will facilitate the identification of cold hardiness to screen existing varieties for cultivation in specific regions and for the breeding of improved varieties.

## 2 Materials and methods

### 2.1 Materials and experimental field

Shoots of experimental materials (20 V*. vinifera* grape varieties) were sampled from an experimental vineyard of the Northwest Agriculture and Forestry University (NWAFU) located in Yangling of Shanxi Province (lat. 34°N, long. 108°E), China. This area has a semiarid continental monsoon climate, and the soil type is bauxite. Self-rooted vines of *V. vinifera*. were planted in 2013. Vine rows were oriented west - east, with vines spaced in 1.0 × 2.5 m rows. The vines were cordon-trained and pruned to two buds per spur. All viticultural practices were performed according to local standards.

### 2.2 Experimental design

Sampling was conducted on January 10, 2021, and 35 vines of each variety with good growth condition were randomly selected. Ten dormant shoots (one-year-old shoots, the number of dormant buds on each shoot is not less than six) were collected for a total of 350 shoots collected from each variety. The collected shoots were rinsed three times with tap water, three times with deionized water, and then the water was adsorbed using filter paper. The shoots were divided in seven plastic bags, with fifty shoots for each variety per bag. Six bags were placed in a chamber with controlled temperature and humidity for freeze-thaw treatment for evaluation of cold hardiness by EL, TTC, and RG methods. By EL and TTC methods, the amounts of shoots treated at each temperature was nine, including three biological replicates and three technical replicates; by the RG method, the amounts of dormant buds treated at each temperature was 90, including three biological replicates and three technical replicates. Samples were freeze-thaw treated in accordance with the method of Zhang et al., with slight modifications (Zhang et al., 2013). Six temperatures were used: 4°C (control temperature treatment), -10°C, -14°C, -18°C, -22°C, and -26°C for the six treatment groups. The freezing treatment lasted 12 h and the recovery period was 4 h at room temperature to thaw the samples. The cooling and heating rates were both set at 4°C.h^-1^. A sample of fifty shoots for each variety was stored in a refrigerator at 4°C and used as test materials were used to evaluate cold hardiness by DTA method and to determine physiological and biochemical indexes related to cold hardiness. by the DTA method, the amounts of one-year-old shoots and dormant buds were performed 54 points, including three biological replicates and three technical replicates.

### 2.3 Evaluation of cold hardiness

#### 2.3.1 Evaluating cold hardiness by EL

EL was performed as described by Wang et al. (2021b). Take three shoots of each variety were prepared for each freezing temperature, remove the epidermis, avoid the buds, select the stems and cut them into 1∽2 mm slices and mix evenly. Samples weighing 2 g were transferred into a 25 ml test tube with a stopper, and then 20 mL deionized water was added and shaken well. This was done three times for each treatment. After shaking for 12 hours in a shaker, a conductivity meter (DDS‐11C, Shanghai optical instrument fac‐ tory, Shanghai, China) was used to determine the initial conductivity value. Each test tube was then boiled for 40 min, allowed to stand for 2 hours, and then the final conductivity value was determined. Experiments were repeated three times with three biological replicates. The relative conductivity was calculated as follows. Relative conductivity (%) = (initial conductivity value/final conductivity value) × 100. LT_50_ value was calculated using the logistic regression equation: Y = K/(1 + ae^−bx^). Y is the relative conductivity, x is the processing temperature, and K is the maximum leakage (K = 100). In practical application: Y´ = ln [(K − Y)/Y]. Y´ = lna − bx, that is, the relative conductivity Y is converted into Y´, and the relation‐ ship between it and the processing temperature is expressed by linearity. The parameter a and b of the equation were obtained by linear regression. The inflection point temperature is the LT_50_.

#### 2.3.2 Evaluating cold hardiness by DTA

Refer to the method of Kaya and Köse, with slight modifications (Kaya and Köse, 2017; Küpe and Köse, 2020). Nine shoots of each variety were randomly selected from the samples that have not been freeze-thaw treated for DTA. Temperature exotherms of shoots, including dormant buds and internodes of shoots, were determined by observing temperature recording for sudden temperature deflections from dormant buds and internodes of shoots. Platinum hardiness temperature needle (Model: PT100, Anwei Jujie Technology Co., Ltd., Wuhu, China) were inserted in the intact dormant buds and internodes of shoots and fixed with elastic band. Silicon grease was used to cover the thermocouple junction to obtain maximum heat transfer. Then the samples were wrapped with aluminium foil and placed in Dewar Flaks which was prechilled to 4°C. Dewar Flaks were taken into a Programmable Temperature & Humidity Chamber (Model: YSGJS-408, Shanghai Lanhao Instrument & Equipment Co., Ltd., Shanghai, China), equipped with a temperature controller, to achieve a constant cooling rate that was 4°C·h^-1^. Cooling started at 4°C in all freezing tests and ended at -26°C. Low temperature exotherms (LTEs) were identified from temperature data recorded at 2^-s^ intervals using 48-channel data acquisition system, Data Acquisition System (Model: R8000, Anwei Jujie Technology Co., Ltd., Wuhu, China) in computer. As the temperature drops, an inflection point appears in the cooling curve. The starting point of the inflection point is the supercooling point, and the peak value is the freezing point. Lethal temperatures for shoot were expressed as LTE_50_ (the temperature of the median LTE’s), the temperatures at which 50% of dormant buds or internodes of shoots were killed. DTA was performed on 18 points (3 repeats, 6 points for each repeat) for an independent experiment between the dormant buds and internodes of shoots and repeated three independent experiments.

#### 2.3.3 Evaluating cold hardiness by TTC

TTC was performed as described by Zhao et al. (2018). After the freeze-thaw treatment, 10 5-mm thick sections were cut from shoot internodes and placed in 10 mL 0.5% TTC in the dark in a 30°C incubator for 4 d. Then, the samples were dissected to obtain longitudinal sections, which were projected onto a LA2400 scanner having image analysis software (Win RHIZO™; Regent Instruments Inc. Quebec City, Quebec, Canada), and the areas stained by TTC on the longitudinal sections were measured. The level of cold injury for each shoot was recorded as one of five levels based on the area of staining of the whole longitudinal section. Level 1 = 0% – 5.0%, 2 = 5.1% – 40.0%, 3 = 40.1% – 70.0%, 4 = 70.1% – 90.0%, 5 = 90.1% – 100% staining area. Then, the staining index was calculated at related levels as follows: Staining index=Σ (staining level × shoot sections at the level)/(4 × total shoot sections). The LT_50_ value was determined using a logistic equation of staining indices. Experiments were repeated three times with three biological replicates.

#### 2.3.4 Evaluating cold hardiness by RG

Refer to the method of Liu Z. et al. (2021) slightly modified. The shoots treated with different low temperatures were soaked in water at room temperature for 12 hours and cut into single-bud stem cuttings. The cross section of the cut was wrapped with plastic film to avoid water loss. The single-bud stem cuttings were inserted into the foam board, and the bottom cross section was exposed 1~3cm, which was put into the test box of 30*20*8 cm with tap water and cultured in the room. Cultured under the light intensity of 2000~3000 Lx for 12 h, in the dark for 12 h, the temperature was 25-28°C. Each variety is divided into 3 parts, each with 30 single-bud cuttings. The dynamics of shoots were observed every day, and the germination rate was counted after 30 days. The formula of germination rate: germination rate (%) = (number of budding shoots/number of cuttings) × 100%. According to the sprouting changes of single bud cuttings of different varieties under low temperature treatments, combined with logistic equation and to determine LT_50_.

### 2.4 Determination of physiological and biochemical indicators

#### 2.4.1 Water contents

Total water (%, TW) in shoots was determined as (fresh weight -dry weight) *100, the dry weight after oven-drying at 90°C for 16 h until a constant weight. Free water (%, FW) was determined using a digital display saccharimeter in accordance with the method of Chen et al. (2014). Bound water (%, BW) = TW – FW. Three shoots (only one bud per shoot) were used for water measurements in each replication.

#### 2.4.2 Osmoregulation substances

Samples of shoots (1.0 g) from all treatment groups were ground in a chilled mortar with 1% (w/v) polyvinylpolypyrrolidone, homogenized with 15 mL of 50 mM potassium phosphate buffer (pH 7.8), then centrifuged at 10,000 × *g* for 15min. The resulting supernatant was used for assays. Soluble protein content was determined by Coomassie brilliant blue method, using bovine serum albumin as a standard (Bradford, 1976). The protein content was detected at 525 nm. Proline content was measured based on the method of Bates et al. (1973). Shoot sample (0.2 g) was placed into 5 mL of aqueous sulfosalicylic acid (3%) and kept in a boiling water bath for 30 min. After the mixture was cooled to room temperature, 2.0 mL supernatant extract was mixed with 2.0 mL ninhydrin and 2 mL acetic acid. Then, the mixture was maintained for 30 min in a boiling water bath and cooled in an ice bath. Next, 5 mL of toluene was added, and the mixture was placed in the dark for 5 h. Readings of the colored product were then taken at 520 nm.

#### 2.4.3 Carbohydrate contents

Collected shoot samples were dried to a constant weight and then crushed for the determination of sugar content. Samples of dry powder (0.5 g) were put into 10 mL centrifuge tubes, 8 mL of 80% ethanol solution was added, and extraction was performed for 30 min in a water bath at 80°C with constant stirring. The solution was cooled to room temperature and then centrifuged at 3500 × *g* for 10 min. The supernatant was transferred to a 25 mL volumetric flask and then 6 mL of 80% ethanol was added to the precipitate to repeat the extraction. The supernatants of three extractions were combined and assayed. Reducing sugar content was measured using the 3,5-dinitrosalicylic acid method to determine the absorbance at 520 nm and calculated based on the standard curve of glucose (Miller, 1959). The soluble sugar content was determined by anthrone colorimetry at 620 nm and calculated according to the standard curve of glucose (Yemm and Willis, 1954).

To measure the sucrose content, a sample (10 mL) of the reducing sugar extract was transferred to a 100 mL Erlenmeyer flask, mixed with 10 mL of 6 mol/L hydrochloric acid, boiled in a water bath for 10 min, titrated with 10% NaOH to neutrality after cooling, and then diluted to 50 mL with water to produce the sucrose extract. This extract was then subjected to the 3,5-dinitrosalicylic acid method to determine the sucrose content. After extracting the reducing sugar, the residue was transferred to a 100 mL Erlenmeyer flask, 10 mL 6 mol/L hydrochloric acid was added and mixed well before boiling in a water bath for 10-30 min (Gao, 2006). After cooling, 20 mL water was added and then the supernatant was filtered into a 50 mL volumetric flask. The residue was washed three times and filtered again, and then the volume was adjusted to 50 mL to obtain the starch extract. The extract was subjected to centrifugation at 4000 × *g* for 10 min and then the supernatant was diluted twice before determination of the starch content using the 3,5-dinitrosalicylic acid method, as was done to measure reducing sugar content (Gao, 2006).

#### 2.4.4 Oxidative stress indices

Shoots (0.1 g) were ground in liquid nitrogen after removing the epidermis and buds, and extracted with 2ml of 5% (w/v) trichloroacetic acid, then centrifuged at 10,000 × *g* for 20 min. The supernatant was used for the assay of H_2_O_2_, as described by Patterson et al. (1984). Superoxide anions ( ${\text{O}}_{2}^{-}$ ) production was estimated as described by Elstner and Heupel (Elstner and Heupel, 1976). After the sample (1 g) was added to the 65 mM phosphate buffer to polish, the mixture was centrifuged for 10 min at 10,000 × *g* before 10 mM hydroxylamine hydrochloride was heated for 20 min at 25°C. After 17 mM amino benzene sulfonamide acid α-naphthylamine was added, the reaction solution was placed in a water bath (30°C) for 30 min and then analyzed with a colorimetric spectrophotometer at 530 nm. Results were compared to standard curves. Malondialdehyde (MDA) content was determined by thiobarbituric acid-reactive substances methods (Hodges et al., 1999). Shoot sample (2.0 g) was homogenized in 15 mL 0.1% TCA and then centrifuged at 5,000 × *g* for 10min. Five milliliters of 5% TCA containing 0.5% TBA were added to 1 mL of the supernatant then incubated in boiling water for 10min. Then the reaction tubes were transferred to ice water to stop the reaction. MDA absorption was measured spectrophotometrically at 450, 532, and 600 nm.

#### 2.4.5 Antioxidant enzymes

Samples of shoot tissues (0.1 g) from all tested varieties were ground in a chilled mortar with 2% (w/v) polyvinylpolypyrrolidone, homogenized with 10 mL of 50 mM potassium phosphate buffer (pH 7.8) containing1 mM EDTA-Na_2_ and 0.3% Triton X-100, then centrifuged at 12,000 × *g* for 20 min. The resulting supernatant was used for enzyme assays. Protein content was determined according to Bradford (Bradford, 1976), using bovine serum albumin as a standard. Superoxide dismutase (SOD; EC 1.15.1.1) activity was estimated by the method of Giannopolittis and Ries and was expressed as units/g FW min (Giannopolites and Ries, 1977). Catalase (CAT; EC 1.11.1.6) activity was evaluated according to Aebi and was expressed as units/g FW min (Aebi, 1984). Peroxidase (POD; EC 1.11.1.7) activity was measured using the method of Rao (1996) and was expressed as units/g FW s.

#### 2.4.6 Antioxidant metabolites

Shoot sample (0.4 g) was ground with a mortar and pestle in 2 mL of 0.5 mM EDTA solution containing 3% trichloroacetic acid and centrifuged at 15,000 × *g* for 10 min at 4°C. The supernatant was used for assays of the levels of ascorbic acid (ASA) and glutathione (GSH). The amount of GSH was evaluated following the method of Monostori et al. and was expressed as mg/g FW (Monostori et al., 2009). The amount of ASA was estimated using the method of Foyer et al. and was expressed as mg/g FW (Foyer and Halliwell, 1976).

### 2.5 Statistical analysis

The standardized data for the substances measured were analyzed with SPSS 17.0 and processed using the subordinative function to evaluate the level of cold hardiness of the range of wild grape germplasm examined here. Evaluation of cold hardiness is based on the evaluation of the various subordinative function indices in the form,


$$Uij=\frac{x_{ij}-x_{j\;min}}{x_{j\;max}-x_{j\;min}}$$

(Positive correlation, including BW, Pro, TSS, CAT, and ASA.)


$$Uij=1-\frac{x_{ij}-x_{j\;min}}{x_{j\;max}-x_{j\;min}}$$

(Negative correlation: including MDA.)

Here, *i* is a particular accession, *j* is a particular index, *Xij* is the testing value of the index *j* of accession *i*, *Xjmin* is the minimum value of index *j* for all accessions, *Xjmax* is the maximum value of index *j* of all accessions, *Uij* is the SF value of accession *i*, and index *j* that relates to cold hardiness.

Microsoft Excel 2013 was used to record and process the original data. Origin 9.0 (OriginLab, Northampton, MA, USA) software was used to fit the logistic equation, and LT_50_ values were obtained. Heat Map with Dendrogram and Correlation Plot were performed using Origin 9.0 software. Descriptive statistics were analyzed *via* SPSS 17.0 (Statistical Product and Service Solutions, Inc., Chicago, IL, USA). Values correspond to the mean interval of three independent experiments. Principal component analysis and weight of cold hardiness were performed using SPSSAU, an online platform for data analysis (https://spssau.com).

## 3 Results

### 3.1 Evaluation of cold hardiness of *V. vinifera* by different methods

#### 3.1.1 Evaluation of cold hardiness of V. vinifera based on EL

As shown in **Table 1**, the relative electrolyte leakage of one-year-old shoots of different varieties increased with the decrease of temperature. The LT_50_ values of the 20 tested *V. vinifera* varieties determined by EL ranged from -19.42°C ~ -11.07°C. Meili, Ecolly, Italian Riesling, and Riesling varieties had LT_50_ values below -17°C: -17.76°C, -19.42°C, -17.16°C, and -17.44°C, respectively. Merlot, Petit Verdot, Chardonnay, Sauvignon Blanc, Cabernet Sauvignon, Syrah, and Gewurztraminer varieties had LT_50_ values higher than -13°C: -11.08°C, -10.58°C, -12.28°C, -11.07°C, -12.23°C, -11.78°C, and -12.31°C, respectively. Granoir, Cabernet Sauvignon, Marselan, Dunkelfelder, Pinot Noir, Viognier, Petit Manseng, Yan73, and Ugni Blanc had LT_50_ values that ranged from -13°C to -17°C.

**Table 1:** Identification of cold resistance in 20 wine grape varieties based on EL.

Varieties	Relative electrolyte leakage rate/%						Regression Equation	R^2^	LT_50_ (°C)
	4°C	-10°C	-14°C	-18°C	-22°C	-26°C			
ECL	0.65	11.69	45.91	13.42	66.22	86.45	y=100/(1 + 66.6863e^0.2365x^)	0.95	-19.42
ML	0.58	13.76	29.13	48.55	77.43	86.22	y=100/(1 + 64.7543e^0.2148x^)	0.95	-17.76
RL	0.86	16.13	31.8	52.77	73.80	86.80	y=100/(1 + 47.6508e^0.2215x^)	0.99	-17.44
IRL	7.45	31.53	40.70	46.65	67.50	74.13	y=100/(1 + 7.7191e^0.1191x^)	0.92	-17.16
PN	0.16	10.81	29.47	56.98	85.44	94.03	y=100/(1 + 182.0898e^0.3094x^)	0.98	-16.82
Y73	0.25	14.14	35.37	67.91	81.70	95.89	y=100/(1 + 122.0340e^0.3001x^)	0.95	-16.01
VON	1.47	22.17	66.17	60.58	91.82	96.02	y=100/(1 + 28.8699e^0.2107x^)	0.99	-15.96
PMS	2.97	27.28	43.43	61.50	75.69	87.00	y=100/(1 + 15.9842e^0.1791x^)	0.99	-15.48
DKF	2.94	29.18	46.48	67.92	74.30	90.39	y=100/(1 + 15.6536e^0.1864x^)	0.89	-14.76
CS	1.03	25.27	57.94	54.16	89.40	95.15	y=100/(1 + 35.4634e^0.2484x^)	0.86	-14.37
GN	1.94	29.16	45.35	76.94	81.67	93.13	y=100/(1 + 21.2318e^0.2168x^)	0.94	-14.09
MSL	0.28	23.92	59.01	77.63	94.90	98.64	y=100/(1 + 91.7622e^0.3362x^)	0.98	-13.44
UB	1.21	30.16	54.46	70.97	91.43	96.21	y=100/(1 + 29.5741e^0.2547x^)	0.93	-13.3
GT	5.03	39.75	60.5	68.59	86.62	92.18	y=100/(1 + 9.1871e^0.1802x^)	0.92	-12.31
CDN	3.95	39.01	56.17	77.41	87.60	93.22	y=100/(1 + 11.1017e^0.196x^)	0.99	-12.28
CF	9.55	42.35	57.50	63.57	84.25	85.54	y=100/(1 + 5.4434e^0.1386x^)	0.89	-12.23
SY	3.48	40.74	62.09	77.84	89.82	95.23	y=100/(1 + 11.9401e^0.2105x^)	0.99	-11.78
MLT	5.57	44.97	67.35	78.58	82.10	95.90	y=100/(1 + 7.9965e^0.1877x^)	0.85	-11.08
SB	27.21	48.25	55.84	60.89	64.73	74.09	y=100/(1 + 2.0604e^0.0653x^)	0.94	-11.07
PVD	3.23	46.65	68.97	87.82	89.8	97.9	y=100/(1 + 11.7764e^0.2332x^)	0.82	-10.58

ML (Meili), ECL (Ecolly), GN (Granoir), CS (Cabernet Sauvignon), MSL (Marselan), DKF (Dunkelfelder), PN (Pinot Noir), MLT (Merlot), VON (Viognier), PVD (Petit Verdot), PMS (Petit Manseng), CDN (Chardonnay), SB (Sauvignon Blanc), IRL (Italian Riesling), CF (Cabernet Franc), RL (Riesling), Y73 (YAN73), SY (Syrah), GT (Gewurztraminer), UB (Ugni Blanc). The relative electrolyte leakage rate of one-year-old shoots are the averages of three independent measurements.

#### 3.1.2 Evaluation of cold hardiness of V. vinifera based on DTA

DTA was also used to evaluate cold hardiness of the tested varieties. As shown in **Table 2**, Ecolly shoots (one-year-old shoots) and winter buds exhibited the lowest supercooling point and freezing point, -11.57°C and -10.97°C respectively for shoots, and -10.72°C and -10.02°C, respectively for winter buds. The shoots and winter buds of Dunkelfelder have the strongest supercooling ability. For shoots, the LT_50_ values for the 20 V*. vinifera* varieties determined by DTA ranged from -11.60°C ~ -8.20°C. Shoots of Meili, Ecolly, Italian Riesling, and Riesling exhibited LT_50_ values lower than -10°C, with -11.20°C, -11.60°C, -11.10°C and -10.10°C respectively. Shoots of Dunkelfelder, Merlot, Petit Verdot, Sauvignon Blanc, Cabernet Sauvignon, Syrah, and Gewurztraminer had LT_50_ values higher than -9°C: -8.20°C, -8.80°C, -8.70°C, -8.60°C, -8.40°C, -8.45°C and -8.35 °C, respectively. For buds, the LT_50_ values determined by DTA were in the range of -10.60°C ~ -6.70°C. Buds of Meili, Ecolly, Italian Riesling, Riesling, and Ugni Blanc had LT_50_ values lower than -9°C: -10.50°C, -10.60°C, -9.80°C, -9.70°C and -9.40°C, respectively. Buds of Merlot, Sauvignon Blanc, Syrah, and Gewurztraminer exhibited LT_50_ values higher than -8°C: -6.70°C, -6.80°C, -7.95°C, and -7.60°C, respectively.

**Table 2:** Identification of cold resistance in 20 wine grape varieties based on DTA.

Varieties	Internodes of shoots		Supercooling capacity	LT_50_ (°C)	Varieties	Dormant buds		Supercooling capacity	LT_50_ (°C)
	Supercooling point	Freezing point				Supercooling point	Freezing point		
ECL	-11.57	-10.97	0.60	-11.60	ML	-10.72	-10.02	0.70	-10.60
ML	-10.92	-10.52	0.40	-11.20	ECL	-10.30	-9.30	1.00	-10.50
IRL	-10.88	-10.48	0.40	-11.10	IRL	-9.74	-9.24	0.50	-9.80
RL	-10.84	-10.14	0.70	-10.90	RL	-9.64	-8.74	0.90	-9.70
UB	-10.18	-9.68	0.50	-10.10	UB	-9.17	-8.77	0.40	-9.40
MSL	-9.76	-9.26	0.50	-10.00	GN	-8.94	-8.44	0.50	-9.00
VON	-9.71	-9.21	0.50	-9.80	VON	-8.88	-8.18	0.70	-8.90
Y73	-9.58	-8.88	0.70	-9.45	PN	-8.28	-7.48	0.80	-8.80
CDN	-9.15	-8.25	0.90	-9.20	PMS	-8.40	-7.50	0.90	-8.80
CS	-9.00	-8.60	0.40	-9.10	Y73	-8.16	-7.06	1.10	-8.60
PN	-9.25	-8.75	0.50	-9.10	CS	-8.13	-7.13	1.00	-8.55
PMS	-9.08	-8.48	0.60	-9.10	CDN	-8.50	-7.90	0.60	-8.50
GN	-9.04	-8.64	0.40	-9.00	MSL	-8.52	-8.02	0.50	-8.40
MLT	-8.84	-8.54	0.30	-8.80	DKF	-8.22	-6.62	1.60	-8.20
PVD	-8.66	-7.96	0.70	-8.70	CF	-7.90	-7.20	0.70	-8.20
SB	-8.60	-8.20	0.40	-8.60	PVD	-7.72	-7.02	0.70	-8.00
SY	-8.35	-7.65	0.70	-8.45	SY	-8.00	-7.10	0.90	-7.95
CF	-8.62	-7.82	0.80	-8.40	GT	-7.80	-7.50	0.30	-7.60
GT	-8.47	-7.87	0.60	-8.35	SB	-6.90	-6.50	0.40	-6.80
DKF	-8.56	-7.36	1.20	-8.20	MLT	-6.70	-5.90	0.80	-6.70

Annotations of varieties same as above. The supercooling point and freezing point are the mean values of three independent experiments and the supercooling capacity is the difference between the supercooling point and the freezing point. The LT_50_ values of one-year-old shoots and dormant buds are the averages of three independent measurements.

#### 3.1.3 Evaluation of cold hardiness of V. vinifera based on TTC

As shown in **Table 3**, the staining index of one-year-old shoots of different varieties decreased with the decrease of temperature. The LT_50_ values of the 20 tested *V. vinifera* varieties determined by TTC ranged from -23.74°C ~ -15.87°C. Meili, Ecolly, Italian Riesling, Riesling, and Ugni Blanc varieties had LT_50_ values below -20°C: -20.31°C, -21.07°C, -23.74°C, -20.48°C, and -20.07°C, respectively. Merlot, Petit Verdot, Cabernet Franc, and Syrah varieties had LT_50_ values higher than -18°C: -16.21°C, -17.57°C, -15.87°C, and -17.98°C, respectively. Granoir, Cabernet Sauvignon, Marselan, Dunkelfelder, Pinot Noir, Viognier, Petit manseng, Chardonnay, Sauvignon Blanc, Yan73, and Gerwurztraminer had LT_50_ values that ranged from -20°C∽-18°C.

**Table 3:** Identification of cold resistance in 20 V*. vinifera* varieties based on TTC.

Varieties	Staining index						Regression Equation	R^2^	LT_50_ (°C)
	4°C	-10°C	-14°C	-18°C	-22°C	-26°C			
IRL	0.79	0.71	0.66	0.58	0.54	0.46	y=100/(1+0.2048e^-0.0668x^)	0.98	-23.74
ECL	0.97	0.83	0.75	0.57	0.46	0.35	y=100/(1+0.0521e^-0.1402x^)	0.97	-21.07
RL	0.95	0.79	0.69	0.58	0.40	0.38	y=100/(1+0.0819e^-0.1222x^)	0.96	-20.48
ML	0.88	0.69	0.63	0.56	0.47	0.38	y=100/(1+0.1950e^-0.0805x^)	0.99	-20.31
UB	0.96	0.80	0.70	0.56	0.45	0.30	y=100/(1+0.0636e^-0.1373x^)	0.98	-20.07
PN	0.85	0.67	0.60	0.54	0.46	0.39	y=100/(1+0.2409e^-0.0716x^)	0.99	-19.88
CDN	0.71	0.60	0.56	0.53	0.46	0.44	y=100/(1+0.4662e^-0.0386x^)	0.97	-19.77
MSL	0.98	0.80	0.75	0.64	0.35	0.27	y=100/(1+0.0398e^-0.1632x^)	0.90	-19.76
Y73	0.99	0.87	0.75	0.60	0.38	0.22	y=100/(1+0.0192e^-0.2012x^)	0.99	-19.64
DKF	0.92	0.71	0.66	0.57	0.40	0.35	y=100/(1+0.1369e^-0.1016x^)	0.96	-19.57
VON	0.95	0.77	0.64	0.57	0.45	0.29	y=100/(1+0.0872e^-0.125x^)	0.96	-19.52
CS	0.88	0.67	0.61	0.60	0.42	0.35	y=100/(1+0.1897e^-0.0856x^)	0.90	-19.42
PMS	0.75	0.65	0.57	0.52	0.47	0.40	y=100/(1+0.3769e^-0.0505x^)	0.97	-19.32
SB	0.91	0.71	0.60	0.56	0.44	0.32	y=100/(1+0.1514e^-0.0983x^)	0.95	-19.21
GN	0.83	0.66	0.59	0.51	0.42	0.29	y=100/(1+0.2390e^-0.0769x^)	0.98	-18.61
GT	0.82	0.71	0.61	0.50	0.40	0.33	y=100/(1+0.2491e^-0.0766x^)	0.96	-18.14
SY	0.87	0.66	0.58	0.50	0.42	0.33	y=100/(1+0.2145e^-0.0856x^)	0.99	-17.98
PVD	0.86	0.64	0.58	0.50	0.44	0.30	y=100/(1+0.2249e^-0.0849x^)	0.96	-17.57
MLT	0.92	0.68	0.57	0.44	0.33	0.23	y=100/(1+0.1370e^-0.1226x^)	0.99	-16.21
CF	0.84	0.63	0.54	0.46	0.35	0.32	y=100/(1+0.2714e^-0.0822x^)	0.97	-15.87

Annotations of varieties same as above. The staining index values of one-year-old shoots are the averages of three independent measurements.

#### 3.1.4 Evaluation of cold hardiness of V. vinifera based on RG

As shown in **Table 4**, the germination rates of buds of different varieties decreased with the decrease of temperature. The LT_50_ values range of 20 tested *V. viniferae* varieties determined by RG ranged from -21.71°C ~ -12.54°C. Meili, Ecolly, Italian Riesling and Riesling varieties had LT_50_ values below -18°C: -19.67°C, -20.71°C, -20.33°C, and -18.78°C, respectively. Granoir, Sauvignon Blanc, Cabernet Franc, Syrah, and Gewurztraminer varieties had LT_50_ values higher than -15°C: -13.11°C, -13.22°C, -12.88°C, -13.98°C, and -12.54°C, respectively. Cabernet Sauvignon, Marselan, Dunkelfelder, Pinot Noir, Merlot, Viognier, Petit Verdot, Petit Manseng, Chardonnay, Yan73, and Ugni Blanc had LT_50_ values that ranged from -15°C to -18°C.

**Table 4:** Identification of cold resistance in 20 V*. vinifera* varieties based on RG.

Varieties	Germination rate/%						Regression Equation	R^2^	LT_50_ (°C)
	4°C	-10°C	-14°C	-18°C	-22°C	-26°C			
ECL	96.82	95.24	70.99	51.16	38.55	0.00	y=100/(1+0.0378e^-0.1581x^)	0.82	-20.71
IRL	100.00	95.24	86.51	66.46	41.61	14.44	y=100/(1+0.0026e^-0.2937x^)	0.99	-20.33
ML	100.00	95.54	87.80	76.58	30.76	7.54	y=100/(1+0.0011e^-0.3481x^)	0.96	-19.67
RL	100.00	93.94	79.68	60.45	26.59	0.00	y=100/(1+0.0028e^-0.313x^)	0.99	-18.78
UB	94.44	76.25	58.61	48.52	35.63	0.00	y=100/(1+0.0967e^-0.1331x^)	0.98	-17.56
CS	100.00	96.06	81.40	43.26	12.95	2.08	y=100/(1+0.0005e^-0.4368x^)	0.99	-17.38
PN	100.00	82.60	67.08	46.64	27.28	6.87	y=100/(1+0.0254e^-0.2115x^)	0.97	-17.36
PMS	97.62	78.23	64.20	37.20	10.84	0.00	y=100/(1+0.0489e^-0.1739x^)	0.96	-17.36
VON	95.56	75.18	74.58	48.23	23.61	0.00	y=100/(1+0.0713e^-0.1533x^)	0.94	-17.23
Y73	100.00	90.79	78.98	43.84	21.09	2.08	y=100/(1+0.0006e^-0.4339x^)	0.99	-17.20
CDN	97.78	78.05	63.41	45.79	39.15	0.00	y=100/(1+0.0466e^-0.1739x^)	0.99	-17.06
MSL	98.41	79.88	63.35	58.05	24.68	8.37	y=100/(1+0.0315e^-0.2079x^)	0.97	-16.63
DKF	97.78	88.00	80.09	40.44	22.89	6.83	y=100/(1+0.0355e^-0.2069x^)	0.93	-16.13
PVD	100.00	91.32	73.98	27.08	6.53	0.00	y=100/(1+0.0015e^-0.4179x^)	0.99	-15.63
MLT	97.62	75.23	57.50	72.65	7.12	0.00	y=100/(1+0.0436e^-0.2022x^)	0.81	-15.50
SY	96.97	78.28	49.87	31.52	17.55	0.00	y=100/(1+0.0676e^-0.1928x^)	0.98	-13.98
SB	100.00	75.98	43.10	25.36	4.17	0.00	y=100/(1+0.0089e^-0.3573x^)	0.97	-13.22
GN	98.48	68.33	44.54	26.8	10.01	3.45	y=100/(1+0.0392e^-0.2471x^)	0.99	-13.11
CF	97.22	65.78	43.71	34.56	2.78	0.00	y=100/(1+0.0540e^-0.2266x^)	0.92	-12.88
GT	96.67	63.14	42.3	44.07	5.81	0.00	y=100/(1+0.0699e^-0.2122x^)	0.92	-12.54

Annotations of varieties same as above. The germination rate of buds are the averages of three independent measurements.

#### 3.1.5 Analysis of results obtained by different evaluation methods

**Figure 1:**
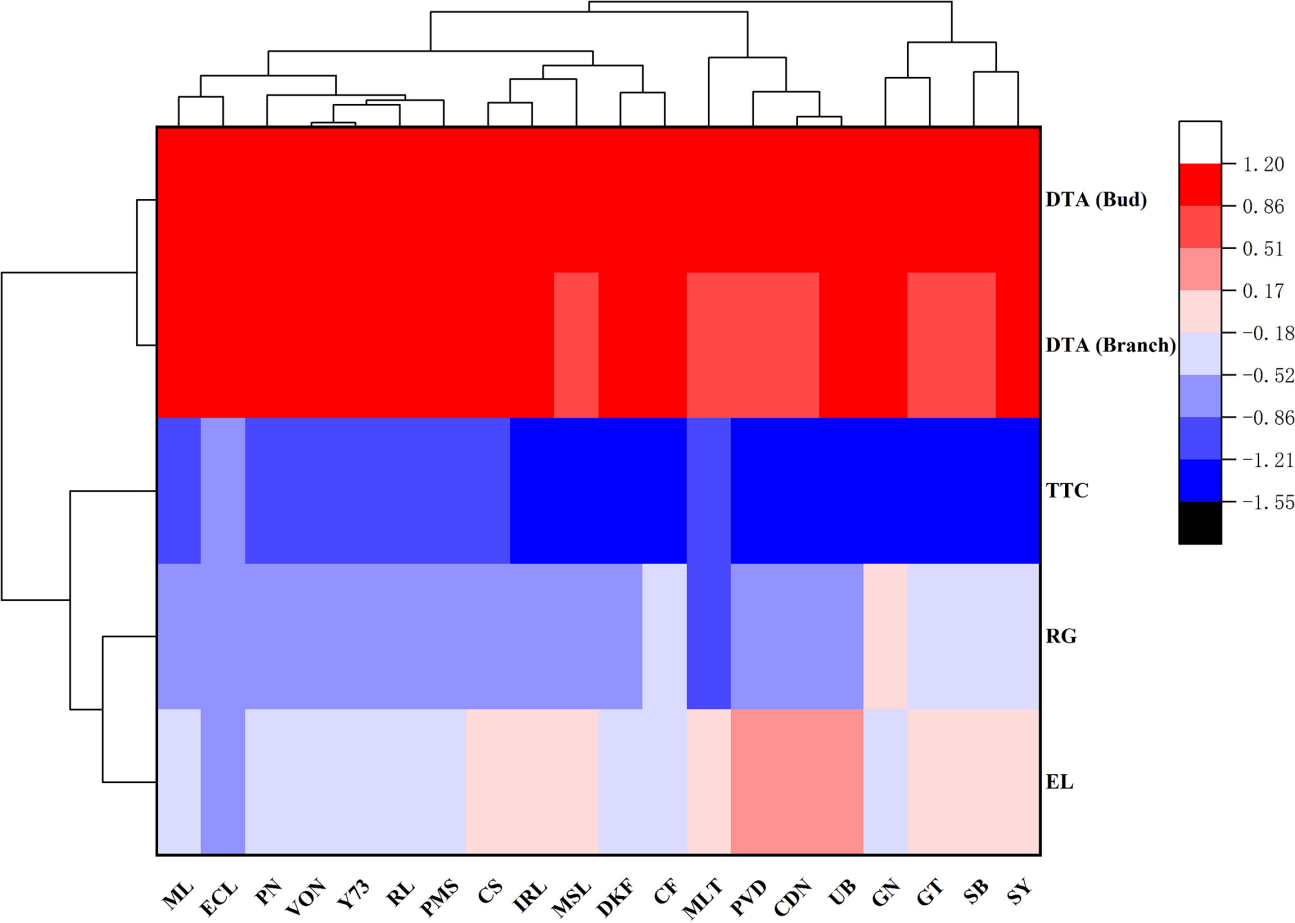
Clustering of LT_50_ values of *V. vinifera* varieties subjected to different evaluation methods. Note: The results (LT_50_ values) of different evaluation methods were quantified by the software SPSS17.0. ML (Meili), ECL (Ecolly), GN (Granoir), CS (Cabernet Sauvignon), MSL (Marselan), DKF (Dunkelfelder), PN (Pinot Noir), MLT (Merlot), VON (Viognier), PVD (Petit Verdot), PMS (Petit Manseng), CDN (Chardonnay), SB (Sauvignon Blanc), IRL (Italian Riesling), CF (Cabernet Franc), RL (Riesling), Y73 (YAN73), SY (Syrah), GT (Gewurztraminer), UB (Ugni Blanc).

The LT_50_ values obtained through the different evaluation methods were analyzed by clustering, and the results are shown in **Figure 1**. The cold hardiness of the 20 tested *V. vinifera* varieties can be clustered into five categories. Meili and Ecolly clustered together with the highest cold hardiness. Pinot Noir, Viognier, Yan73, Riesling, and Petit Manseng clustered together with cold hardiness. Cabernet Sauvignon, Italian Riesling, Marselan, Dunkelfelder, and Cabernet Franc clustered together with moderate cold hardiness. Merlot, Petit Verdot, Chardonnay, and Ugni Blanc clustered together with low cold hardiness. Granoir, Gewurztraminer, Sauvignon Blanc, and Syrah clustered together with the lowest cold hardiness. The four evaluation methods can be divided into three categories: DTA (shoots and buds) and TTC clustered into one group, and EL and RG clustered into separate groups.

**Figure 2:**
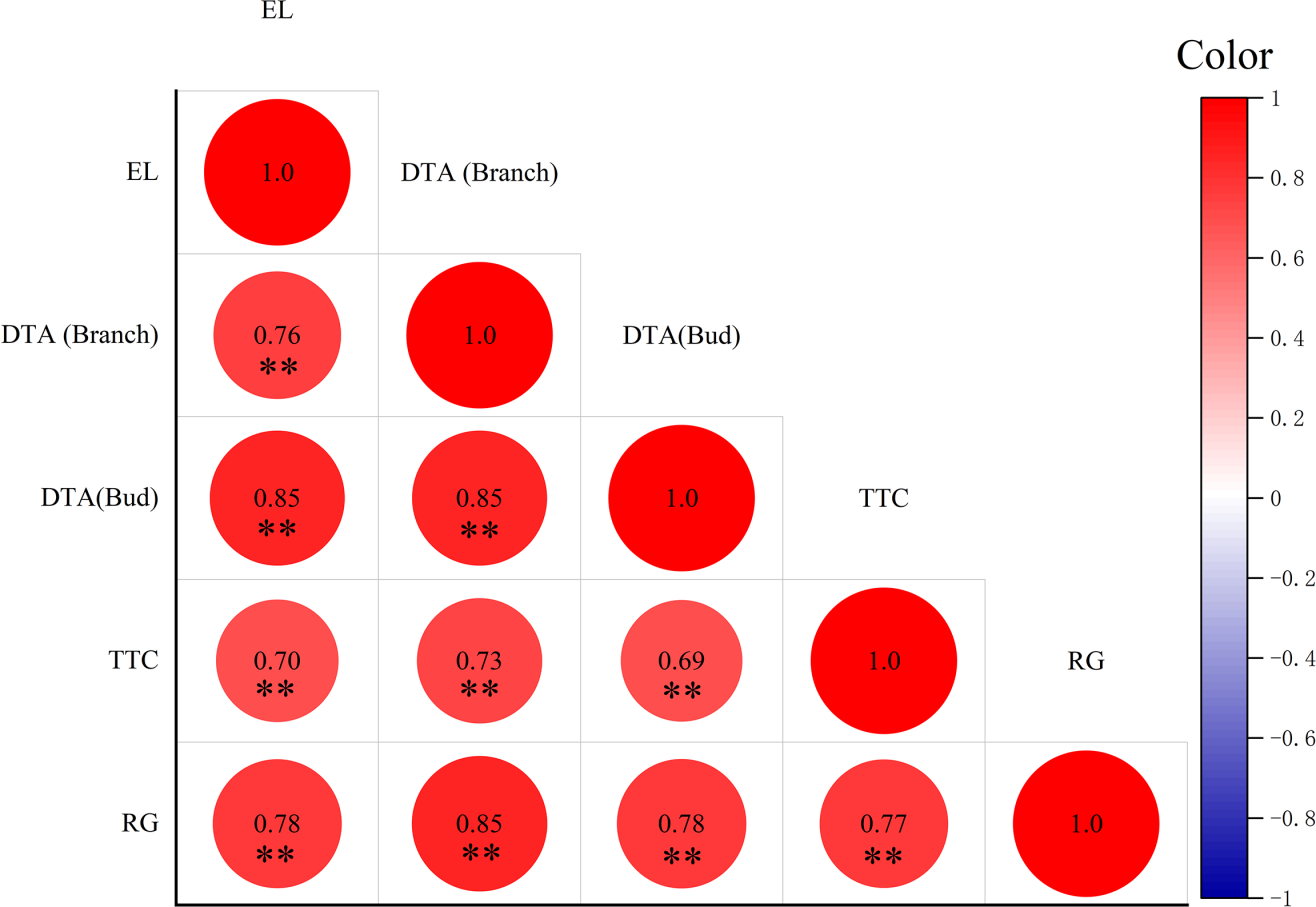
Correlation of LT_50_ values estimated by different methods. Data were tested by Student’s t-test, **p < 0.01 represent significant differences between methods. The larger the circle, the larger the correlation coefficient. Red is positive, blue is negative.

Correlation analysis was performed of the different evaluation methods and the results are shown in **Figure 2**. There was a positive correlation among different evaluation methods, and all reached a very significant level. The correlation coefficients of DTA measured in shoots with DTA measured in buds, TTC, RG, and EL were 0.76, 0.85, 0.70 and 0.78 respectively. The correlation coefficients between bud DTA and TTC, RG, and shoot DTA measurements were 0.85, 0.73, and 0.85 respectively. The correlation coefficients between TTC and RG and DTA of buds were 0.69 and 0.78 respectively. The correlation coefficient between RG and TTC was 0.77.

### 3.2 Analysis and screening of cold hardiness indexes

#### 3.2.1 Correlation analysis of cold hardiness indexes

**Figure 3:**
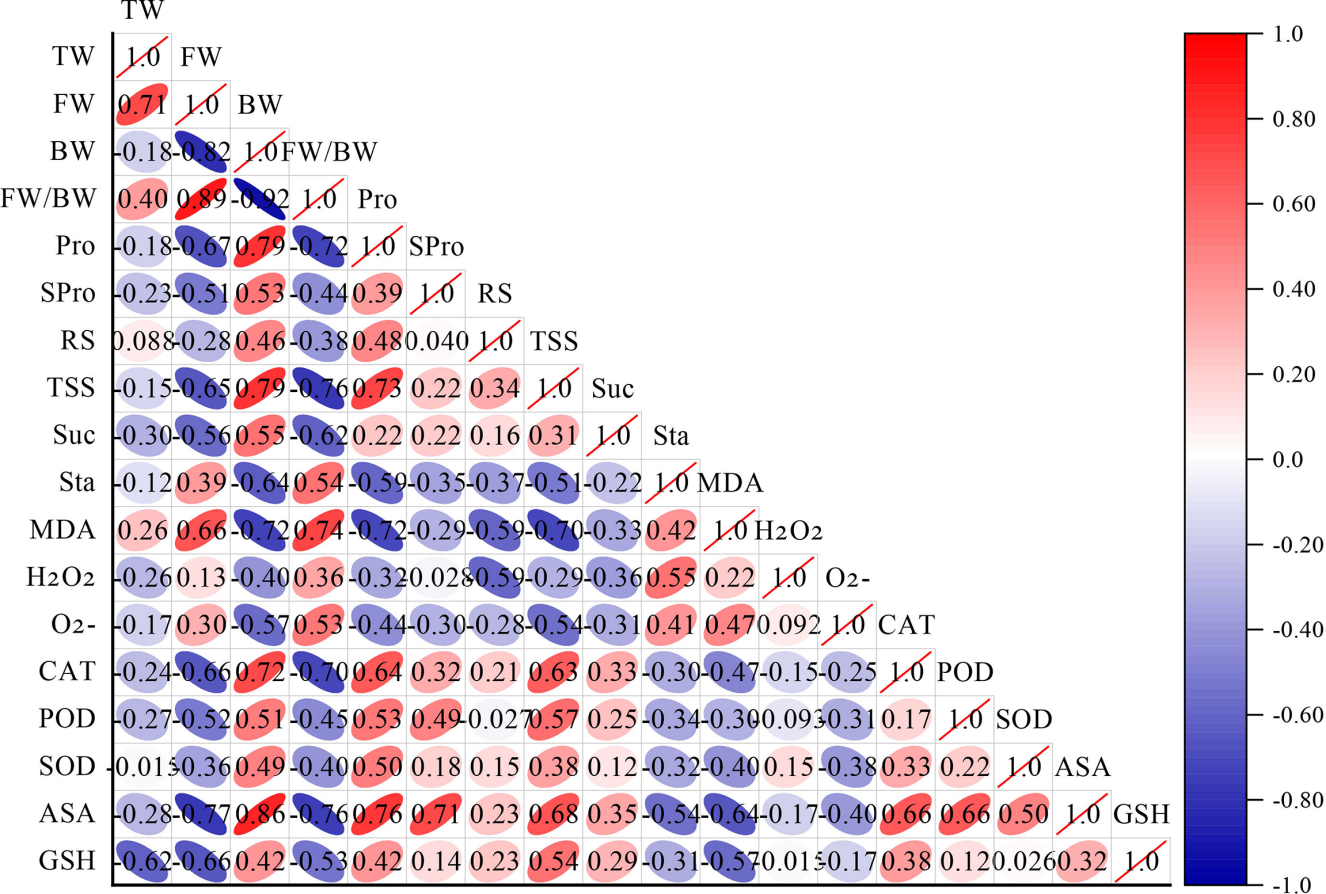
Correlation analysis of cold resistance indexes of different *V. vinifera* varieties. The more slender the ellipse, the larger the correlation coefficient. Red is positive, blue is negative. TW (Total water content), FW (Free water content), BW (Bound water content), FW/BW (The ratio of FW/BW), Pro (Proline content), SPro (Soluble protein content), RS (Reducing sugar content), TSS (Total soluble sugar content), Suc (Sucrose content), Sta (Starch content), MDA (Malondialdehyde content), H_2_O_2_ (hydrogen peroxide content), ${\text{O}}_{2}^{-}$ (Superoxide anions content), CAT (Catalase content), POD (Peroxidase content), SOD (Superoxide dismutase content), ASA (Ascorbic acid content), GSH (Reduced glutathione content).

As shown in **Figure 3**, correlation analysis of the cold hardiness indicators revealed a high positive correlation between free water content (FW) and total water content (TW). The ratio of free water to bound water (FW/BW) was highly positively correlated with FW. Proline content (Pro) was highly positively correlated with bound water (BW). Total soluble sugar content (TSS) was highly positively correlated with BW and Pro. Malondialdehyde content (MDA) was highly positively correlated with FW and FW/BW. Catalase content (CAT) was highly positively correlated with BW, Pro, and TSS. Ascorbic acid content (ASA) was highly positively correlated with BW, Pro, soluble protein content (SPro), TSS, catalase (CAT), and peroxidase content (POD). BW was highly negatively correlated with FW. FW/BW was highly negatively correlated with BW. Pro was highly negatively correlated with FW and FW/BW. TSS was highly negatively correlated with FW and FW/BW. Sucrose content (Suc) was highly negatively correlated with FW/BW. Starch content (Sta) was highly negatively correlated with BW. MDA was highly negatively correlated with BW, Pro, and TSS. CAT was highly negatively correlated with FW and FW/BW. ASA was highly negatively correlated with FW, FW/BW, and MDA. Reduced glutathione content (GSH) was highly negatively correlated with TW and FW.

#### 3.2.2 Selection and weight of cold hardiness index

**Figure 4:**
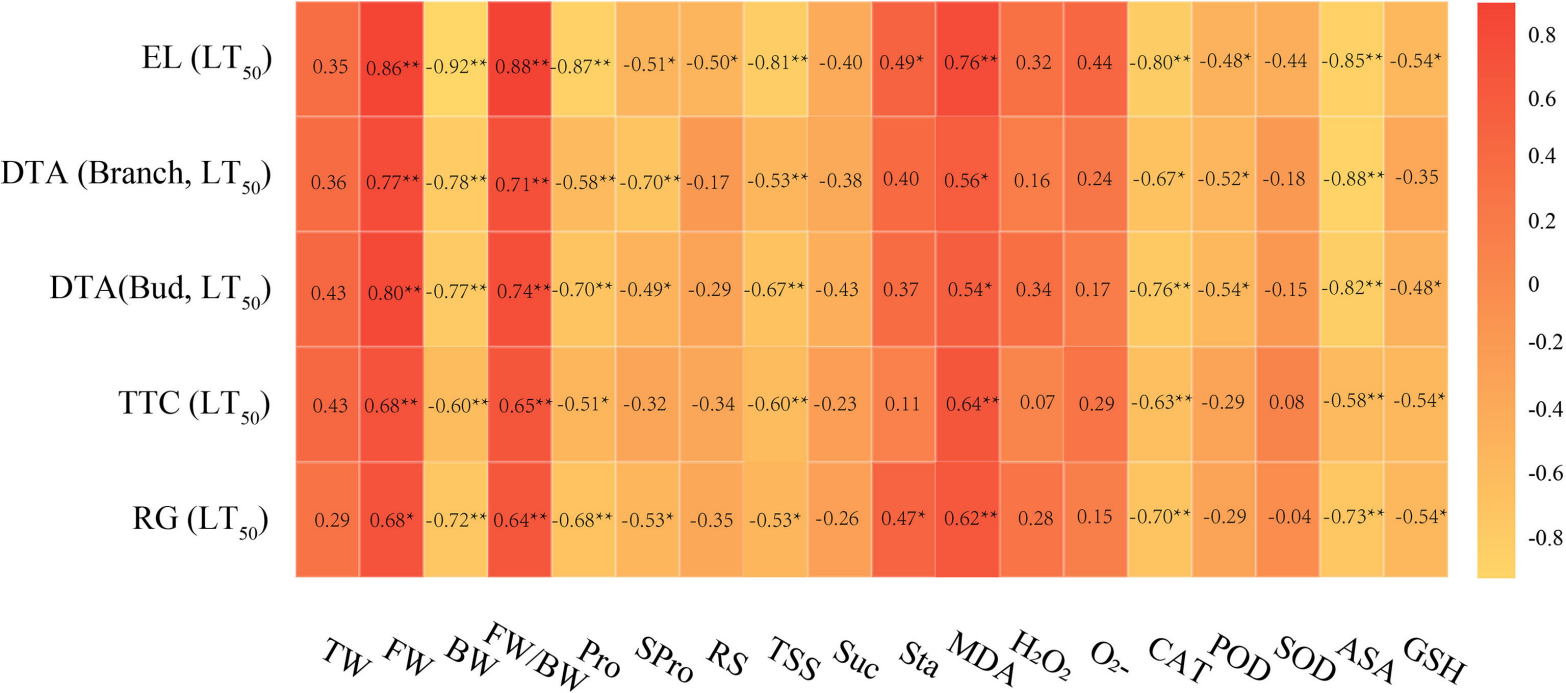
Correlation analysis of LT_50_ values and cold resistance indicators. Data were tested by Student’s t-test, **p < 0.01 represent significant differences between LT_50_ values measured by different evaluation methods and different cold resistance indicators.

Correlation analysis of LT_50_ values measured by different evaluation methods and different cold hardiness indicators are shown in **Figure 4**. The amounts of free water (FW), bound water (BW), proline (Pro), total soluble sugar (TSS), malondialdehyde (MDA), catalase (CAT), and ascorbic acid (ASA), and, as well as the ratio of free water to bound water (FW/BW) of grapevine were significantly correlated with LT_50_ values measured by different evaluation methods. Among the water indexes, FW and LT_50_ values showed a high correlation coefficient overall, with a negative correlation. For osmoregulatory substances, Pro and LT_50_ values showed high overall correlation with a negative correlation. Among carbohydrate indexes, TSS and LT_50_ values were highly and negatively correlated. For the oxidative metabolites, MDA and LT_50_ values showed a high positive correlation coefficient. Of the antioxidant enzymes, CAT and LT_50_ values showed a high negative correlation coefficient. Among the antioxidant metabolites, ASA and LT_50_ values showed a high negative correlation coefficient. The high degree of correlation suggests that BW, Pro, TSS, MDA, CAT, and ASA can be used as primary indicators to evaluate cold hardiness in *V. vinifera*.

The six cold hardiness indexes were standardized and then make principal component analysis (PCA) was performed. The results are shown in **Table 5**. PCA extracted one principal component, with characteristic root value greater than 1. The variance explanation rate of this one principal component was 75.274%, so the cumulative variance explanation rate was 75.274%. This single component reflects most grapevine cold hardiness information. To confirm the result, the principal component was removed from this analysis, and its corresponding weighted variance explanation rate is 75.274/75.274 = 100.00%.

**Table 5:** The result of PCA.

Component	Characteristic root	Variance explanation ratio (%)	Cumulative rate (%)	Principle component extraction		
				Characteristic root	Variance explanation ratio (%)	Cumulative rate (%)
1	4.516	75.274	75.274	4.516	75.274	75.274
2	0.551	9.179	84.452			
3	0.338	5.627	90.079			
4	0.245	4.092	94.170			
5	0.235	3.913	98.084			
6	0.115	1.916	100			

**Table 6** shows the extracted information of cold hardiness index by principal component, and the corresponding relationship between the principal component and the cold hardiness index. The communalities of all cold hardiness indexes are higher than 0.4, indicating strong correlation between indexes and principal components so principal components can be used to effectively extract information. Component score 1 = 0.208*BW + 0.198*Pro + 0.192*TSS – 0.181*MDA + 0.174*CAT + 0.197*ASA. The indexes that influence grape cold hardiness from strongest to weakest are BW, Pro, ASA, TSS, MDA, and CAT, and the corresponding weights are 18.08%, 17.22%, 17.09%, 16.73%, 15.71%, and 15.16%, respectively.

**Table 6:** Principal component analysis of cold resistance indexes.

Indexes	Loading coefficient	Communalities	Component Score Coefficient Matrix	Composite score coefficient	Weight
	Prin1		Prin1		
BW	0.940	0.883	0.208	0.4422	18.08%
Pro	0.895	0.801	0.198	0.4212	17.22%
TSS	0.869	0.756	0.192	0.4091	16.73%
MDA	-0.816	0.667	-0.181	-0.3842	15.71%
CAT	0.788	0.621	0.174	0.3708	15.16%
ASA	0.888	0.789	0.197	0.4179	17.09%

### 3.3 Establishment of comprehensive evaluation method of cold hardiness

#### 3.3.1 Subordination function method and comprehensive evaluation of grapevine cold hardiness index

The different indexes have different units, nature, and quantity, making it necessary to carry out standard quantification. The change of each index is continuous, so the subordination function method (SF) can be used for standard quantification. The ascending and descending order of the subordination function is determined according to the positive and negative loading of the principal component factors. According to the subordination function value and weight of each index, the addition and multiplication rule Ci= Wij × R(Xij) can be applied, where Wij is the weight of each index and R (Xij) is the subordination function value of each index. By the calculation of each index, the comprehensive index of cold hardiness of grape varieties (Ci) was obtained, as shown in **Table 7**. This comprehensive index of cold hardiness was then applied, and the order of cold hardiness of different varieties is as follows: Ecolly > Meili > Viognier > Riesling > Petit Manseng > Italian Riesling > Yan73 > Pinot Noir > Dunkelfelder > Marselan > Cabernet Sauvignon > Granoir > Ugni Blanc > Gewurztraminer > Chardonnay >Syrah > Cabernet Franc > Sauvignon Blanc > Petit Verdot > Merlot.

**Table 7:** Subordination values of cold resistance indicators of shoots from different varieties.

Varieties	BW	Pro	TSS	MDA	CAT	ASA	Composite index
ECL	0.1711	1.0000	1.0000	0.8850	0.7115	1.0000	0.7884
ML	0.0000	0.7769	0.7605	0.8757	0.9615	0.7772	0.6831
VON	0.3231	0.8123	0.6972	0.9033	0.9231	0.5038	0.6774
RL	0.2748	0.5541	0.6490	0.9352	0.8269	0.7479	0.6539
PMS	0.5324	0.5742	0.7384	0.6093	1.0000	0.2471	0.6084
IRL	0.3468	0.4777	0.6241	1.0000	0.7308	0.5241	0.6070
Y73	0.3295	0.4672	0.6411	0.9238	0.8654	0.3389	0.5817
PN	0.4309	0.6611	0.5723	0.6575	0.7479	0.3651	0.5667
DKF	0.5049	0.4899	0.7434	0.9817	0.3269	0.0658	0.5152
MSL	0.4109	0.1320	0.7183	0.7186	0.6923	0.3869	0.5013
CS	0.5033	0.5860	0.4718	0.6630	0.5769	0.1265	0.4842
GN	0.5108	0.1581	0.7785	0.3420	0.5192	0.1585	0.4095
UB	0.6277	0.1612	0.0925	0.5165	0.8077	0.2190	0.3978
GT	0.7027	0.1617	0.3795	0.8047	0.0192	0.1606	0.3752
CDN	0.6033	0.2563	0.4295	0.4931	0.2885	0.0015	0.3466
CF	0.6201	0.3126	0.1098	0.4375	0.2436	0.1336	0.3129
SB	0.7775	0.1666	0.1393	0.3685	0.1859	0.0415	0.2858
SY	0.6509	0.0193	0.1797	0.0000	0.5423	0.0929	0.2492
PVD	1.0000	0.0000	0.0000	0.1920	0.1731	0.0000	0.2372
MLT	0.6259	0.0470	0.0050	0.3683	0.0000	0.0623	0.1906

#### 3.3.2 Correlation analysis between the composite index and LT_50_ values calculated by different methods

LT_50_ is the main ecological factor characterizing plant cold hardiness. Correlation analysis between the composite index and LT_50_ measured by different evaluation methods is shown in **Table 8**. The composite index showed a very significant negative correlation with the five sets of LT_50_ measurements using the four evaluation methods. The composite index is most highly correlated with the LT_50_ values measured by EL, with a correlation coefficient of -0.938. The correlation coefficients between the composite index and DTA measurement of shoots and buds are -0.738 and -0.808 respectively. The correlation coefficients between the composite index and LT_50_ values measured by TTC and RG are -0.709 and -0.763, respectively.

**Table 8:** Correlation analysis between the cold composite index and LT_50_ values.

Item	EL (LT_50_)	DTA (LT_50_ of shoot)	DTA (LT_50_ of bud)	TTC (LT_50_)	RG (LT_50_)
Composite index	-0.938**	-0.738**	-0.808**	-0709**	-0.763**

Data were tested by Student’s t test, **p < 0.01 represent significant differences between methods.

## 4 Discussion

### 4.1 Evaluation of cold hardiness in *V. vinifera* using different methods

EL is the classical method to evaluate cold hardiness and was used here to screen several varieties (Sutinen et al., 1992). The results showed that cold hardiness was in the order of Ecolly > Meili > Riesling > Italian Riesling > Pinot Noir > Yan73 > Viognier > Petit Manseng > Dunkelfelder > Cabernet Sauvignon > Granoir > Marselan > Ugni Blanc > Gewurztraminer > Chardonnay > Sauvignon Blanc > Syrah > Merlot > Cabernet Franc > Petit Verdot. This order is basically consistent with that of previous work to measure cold hardiness, but the measured LT_50_ values are lower than other published values (Chen et al., 2020b; Wang et al., 2021b). This difference may be related to specifics of our sampling location or sampling time. There is a certain correlation between the cold hardiness of grapevines and the degree of dormancy and the local temperature during overwintering. In the northeast region of China, the cold hardiness of *V*. *amurensis* increased in October, was highest in November, and then gradually decreased (Zhao et al., 2020).

DTA, also known as low temperature exotherm (LTE), depends on the exothermic process when plant tissue freezes at low temperature (Kaya and Köse, 2017). Cold hardiness can be evaluated by detecting and recording the exothermic heat released by DTA (Mills et al., 2006). Median LTE temperatures approximate field temperatures that are lethal to 50% of buds (LT_50_), and thus DTA has become a preferred technique to characterize grapevine cold hardiness (Kaya and Köse, 2017). In this study, we collected the LTE of shoots and buds by DTA. The cold hardiness measurements of the two different parts (LT_50_ of shoot and LT_50_ of bud) were highly correlated, with stronger cold hardiness of shoots than that of buds; this is consistent with the results of previous studies (Chen et al., 2020a). Cold hardiness can vary in different parts of grapevine or during different growth degrees, with the strongest cold hardiness typically seen in shoots, followed by that in the main roots and the secondary roots (Chen et al., 2020a). The frost hardiness of unopened buds is relatively stronger, followed by opened buds, and then by young sprouts (Duan et al., 2017).

There can be significant observational error using TTC, as it is difficult to accurately visualize and quantify the results (Tian et al., 2013). The visual evaluation of a TTC stained image combined with application of the logistic equation is a simple and reliable method for the evaluation of grapevine cold hardiness (Zhao et al., 2018). In this study, the TTC measurements and use of the logistic equation allowed determination of cold hardiness in the following order: Italian Riesling > Ecolly > Riesling > Meili > Ugni Blanc > Pinot Noir > Chardonnay > Marselan > Yan73 > Dunkelfelder > Viognier > Cabernet Sauvignon > Petit Manseng > Sauvignon Blanc > Granoir > Gewurztraminer > Syrah > Petit Verdot > Merlot > Cabernet Franc. This differs from the previous ranking results (Wang et al., 2021b), and the variation may be experimental error related to the easy accumulation of triphenyl formazan in the cross section during the test (Zhao et al., 2018). Overall, the accuracy of this method may not be the highest, but the correlation analysis indicates that this method is sufficiently effective to evaluate the cold hardiness of grapevine.

RG is a more intuitive method in which the germination rate, rooting rate, and callus ratio of shoots are measured to ask how well plants survive after being frozen (Cao et al., 2010). Here, we used the germination rate of cuttings at different low temperatures combined with the logistic equation to provide quantifiable evaluation results for RG. The RG classification has high correlation with the results of other evaluation methods, indicating that RG combined with the logistic equation can be used as a quantitative method for the evaluation of grapevine cold hardiness.

LT_50_ value is used as an indicator of plant stress injury and has been widely applied to measure cold hardiness in grapevine (Zeng et al., 2017; Yang et al., 2020). The clustering results of LT_50_ values determined by different evaluation methods indicated that Meili and Ecolly varieties exhibit the highest cold hardiness; Pinot Noir, Viognier, Yan73, Riesling, and Petit Manseng show cold hardiness; Marselan, Dunkelfelder, Italian Riesling, Cabernet Sauvignon, and Cabernet Franc exhibit moderate cold hardiness; Merlot, Petit Verdot, Chardonnay, and Ugni Blanc show low cold hardiness; and Granoir, Gewurztraminer, Sauvignon Blanc, and Syrah exhibit the lowest cold hardiness. These results are not completely consistent with previous assessments. Ecolly is widely cultivated without soil burial for over-wintering in many areas where this practice is required for other varieties, suggesting this variety and related hybrid breeding technology may help solve this important challenge to viticulture in China. In clustering of 124 V*. vinifera* by Wang et al., Ecolly is in hardiness zone 1, Meili, Italian Riesling, Riesling, Cabernet Sauvignon, Dunkelfelder, and Petit Manseng belong to hardiness zone 2, Ugni Blanc, Marselan, Yan73, Sauvignon Blanc, Petit Verdot Chardonnay, and Pinot Noir belong to hardiness zone 3, Gewurztraminer, Cabernet Franc, and Merlot belong to hardiness zone 4 (Wang et al., 2021b). The differences between the two sets of results may be related to the number of germplasm resources tested and the range of LT_50_ values that characterize the cold hardiness of *V. vinifera*. The cold hardiness of *V. vinifera* is generally poor and the range of LT_50_ values was relatively small, with LT_50_ values determined by EL that are between -22°C∽-13°C (Wang et al., 2021b).

The correlation analysis of the LT_50_ values calculated using the four evaluation methods showed very significant positive correlations among the different evaluation methods. This suggests that EL, DTA, TTC, and RG methods can all be used to evaluate cold hardiness and distinguish the cold hardiness of different varieties. However, for practical measurement, these methods rely on different basic principles, so there are advantages and disadvantages of each approach. During measurement, EL is easily affected by temperature, soaking time, and the dissolution of CO_2_ in the air, leading to poor repeatability of the results (Su et al., 2015a). EL is also time-consuming and requires significant materials (Jiang et al., 1999). As a non-destructive evaluation method of cold hardiness, DTA is vulnerable to any changes in the external environment or altered states of dormancy of plants under natural conditions, decreasing the accuracy of the measured results (Chai et al., 2015). TTC can easily be affected by human subjectivity when visually measuring the degree of browning and coloring, which leads to a large error. To achieve good repeatability, the operator must have proficient experimental skills, as it is difficult for beginners to obtain accurate results (Guo et al., 2018). As an intuitive method to evaluate the cold hardiness of grapevine, RG is limited by requiring a long test period and a large amount of materials (Liu Z. et al., 2021). The germination rate of grapevine is also affected by the water retention of buds, which can be another source of variation (Wang et al., 2020). In practical application, the appropriate evaluation method of cold hardiness should be selected according to practical needs.

### 4.2 Correlation of the cold hardiness index with cold hardiness in *V. vinifera*

Cold hardiness of grapevine is a quantitative characteristic controlled by multiple genes (Wang et al., 2020; Wang Z. L. et al., 2021). Given this, it makes sense that evaluation using a single index is not sufficiently comprehensive to accurately reflect cold hardiness in grapevine (Zhang J. et al., 2012). In this study, 18 physiological and biochemical indexes of cold hardiness were determined, and the correlations between different indexes and LT_50_ values determined by EL were analyzed. The FW, BW, FW/BW, Pro, TSS, MDA, CAT, and ASA of dormant shoots were significantly correlated with the LT_50_ values, suggesting these physiological and biochemical factors are sufficient to evaluate cold hardiness of *V. vinifera* during overwintering. This conclusion is not completely consistent with previous research results. Zhao et al. studied the correlation between physiological and biochemical indexes of *V. amurensis* and the LT_50_ values measured by EL and found significant correlation of FW, BW and TSS with LT_50_ values (Zhao et al., 2020). Su et al. examined the correlation between physiological and biochemical indexes of *V. amurensis* and *V. vinifera* and the LT_50_ values measured by EL and found that TSS, SPro, MDA, and Pro were significantly correlated with LT_50_ measurements (Su et al., 2015b). Cao et al. analyzed the correlation between physiological and biochemical indexes of rootstock, *V. labrusca*, *V. vinifera*, and wild species and found that TSS, Spro, MDA, and Pro exhibited significant correlation with the EL-based LT_50_ values (Cao et al., 2010). The different findings reported by different groups may vary due to the use of different populations of tested materials. This study focused on *V. vinifera*, so the cold hardiness model developed here may be most suitable for the evaluation of cold hardiness of this species.

Further analysis of the correlation between different indexes shows a high correlation between different indexes, which is consistent with previous research (Zhang J. et al., 2012; He, 2015; Zhao et al., 2020). This shows that the information reflected by these cold hardiness indexes is super-imposed, so one or two indexes cannot be used alone to evaluate the cold hardiness of grapevine. Instead, SF should be adopted to more accurately evaluate the cold hardiness of different germplasm resources. Zhang et al. comprehensively evaluated the cold hardiness of 25 wild grape varieties by SF (Zhang J. et al., 2012). Su et al. established a comprehensive evaluation method of cold hardiness through PCA and SF, showing that the average subordinate function value obtained by SF could be used as a comprehensive evaluation index of cold hardiness of *V. amurensis* germplasm (Su et al., 2015b). Accurate evaluation of cold hardiness of *V. vinifera* is essential for cold hardiness breeding using *V. vinifera* as parents.

The acquisition of plant cold hardiness is a complex physiological response process, with contributions from many factors altering cold acclimation capabilities (Strimbeck et al., 2015). In response to low temperature stress, plants will launch a series of signal transduction reactions, the tissue water content decreases, osmoregulative substances accumulate, and antioxidant enzyme activity levels change to increase the cold-hardiness capabilities (Chinnusamy et al., 2007). The water content and state of water in plants significantly determine the cold hardiness of plants. The freezing point of FW is 0°C, but the freezing point of BW is -20°C ~ -25°C. At a low ratio of FW/BW, the freezing temperature will decrease correspondingly, which increases frost hardiness (Wu, 2011). Under cold stress, the increase of SPro can further increase the proportion of bound intracellular water. SPro can also regulate the expression of cold hardiness genes to enhance a plant’s cold hardiness (Dionne et al., 2001). Pro helps maintain osmotic equilibrium between the symplasts and apoplasts, so helps prevent low temperature damage by maintaining the functional integrity of the membrane (Dionne et al., 2001). Sugar can improve the cold hardiness of plants by increasing the concentration of cell sap and lowering the freezing point (Cao et al., 2010). Under cold stress, polysaccharides are hydrolyzed to soluble sugars that increase the osmotic potential of the cytoplasm and lower the freezing temperature (Zhang J. et al., 2012). Generally, cold stress can initiate the accumulation of reactive oxygen species (ROS) such as superoxide radical ( ${\text{O}}_{2}^{-}$ ), hydroxyl radical (OH^-^), and hydrogen peroxide (H_2_O_2_) to increase oxidative stress in the plant (Bajguz and Hayat, 2009; Liu et al., 2011). To protect against low temperature-induced oxidative damage, all plants have developed processes to scavenge ROS by enzymatic antioxidant techniques such as POD, SOD and CAT, as well as non-enzymatic methods (Lee and Lee, 2000). SOD scavenges toxic superoxide radicals and catalyzes the reduction of two superoxide anions into H_2_O_2_ and O_2_ (Miyake and Yokota, 2000). POD is another antioxidant enzyme that converts H_2_O_2_ into O_2_ and H_2_O (Sudhakar et al., 2001). ASA and GSH are important nonenzymatic antioxidants in plant cells. They can directly scavenge ${\text{O}}_{2}^{-}$ and reduce the level of H_2_O_2_ (Meng et al., 2014). In this study, according to the mechanism of the comprehensive cold hardiness index, BW of the water indexes, Pro of the osmoregulation substances, TSS of the carbohydrate indexes, MDA of the oxidation metabolites, CAT of the antioxidant enzyme system, and ASA of the antioxidant metabolites are the main indexes of cold hardiness of *V. vinifera*. These six indexes were given weight in cold hardiness by PCA, allowing the construction of the comprehensive evaluation method of *V. vinifera* by SF. The correlation was analyzed between the composite index and the LT_50_ values calculated using the other evaluation methods. The results showed that there was a very significant negative correlation between the composite index and the LT_50_ values measured by different evaluation methods. The composite index has the highest correlation with the LT_50_ values determined by EL, with a coefficient of -0.938. The results show the comprehensive evaluation method of grapevine cold hardiness constructed by PCA combined with SF can accurately evaluate the cold hardiness of *V. vinifera* germplasm resources.

## 5 Conclusions

Our results showed that Ecolly and Meili have the highest cold hardiness among the 20 V*. vinifera* germplasms tested and can be used as parent materials for cold hardiness breeding. EL, DTA, TTC, and RG can be used to screen cold hardiness of *V. vinifera* and can distinguish the cold hardiness of different varieties. During overwintering, measurements of BW, Pro, TSS, MDA, CAT, and ASA in shoots can be used as the main cold hardiness indexes of *V. vinifera* germplasm. The comprehensive evaluation method constructed by PCA combined with SF can be accurately applied for the evaluation of cold hardiness of *V. vinifera* germplasm resources.

## Data availability statement

The original contributions presented in the study are included in the article/supplementary material. Further inquiries can be directed to the corresponding authors.

## Author contributions

Conceptualization, Z-LW, DW, HW and HL; Data curation, Z-LW, DW, XC and YW; Formal analysis, MH and XH; Funding acquisition, HW and HL; Methodology, FY and Y-HL; Project administration, HW and HL. All authors have read and agreed to the published version of the manuscript.

## Funding

This work was supported by the National Key Research and Development Project (2019YFD1002500), the Key Research and Development Project of Shaanxi Province (2020ZDLNY07-08), the Ningxia Hui Nationality Autonomous Region Major Research and Development Project (2020BCF01003), the Research and application of key technologies for sustainable development of wine industry (LYNJ202110), and the Science and technology plan major project of Yinchuan, Ningxia (YCKJ2020-ZD04).

## Acknowledgments

We would like to thank Rachel M for improving the English in this paper.

## Conflict of interest

The authors declare that the research was conducted in the absence of any commercial or financial relationships that could be construed as a potential conflict of interest.

The reviewers YJ and YW declared a shared affiliation with the authors to the handling editor at the time of review.

## Publisher’s note

All claims expressed in this article are solely those of the authors and do not necessarily represent those of their affiliated organizations, or those of the publisher, the editors and the reviewers. Any product that may be evaluated in this article, or claim that may be made by its manufacturer, is not guaranteed or endorsed by the publisher.
